# An Updated View on an Emerging Target: Selected Papers from the 8th International Conference on Protein Kinase CK2

**DOI:** 10.3390/ph10020033

**Published:** 2017-03-23

**Authors:** Joachim Jose, Marc Le-Borgne, Lorenzo A. Pinna, Mathias Montenarh

**Affiliations:** 1 PharmaCampus, Institute of Pharmaceutical and Medicinal Chemistry, Westfälische Wilhelms-Universität Münster, Corrensstr. 48, 48149 Münster, Germany; 2 EA 4446 Bioactive Molecules and Medicinal Chemistry, Faculté de Pharmacie—ISPB, Université Lyon 1, Université de Lyon, SFR Santé Lyon-Est CNRS UMS3453—INSERM US7, 69373 Lyon cedex 8, France; marc.le-borgne@univ-lyon1.fr; 3 Department of Biomedical Sciences, University of Padova, Via Ugo Bassi 58B, 35131 Padova, Italy; lorenzo.pinna@unipd.it; 4 Medizinische Biochemie und Molekularbiologie, Faculty of Medicine, Saarland University Geb. 44/45, D-66424 Homburg, Germany; Mathias.Montenarh@uks.eu

The 8th International Conference on Protein Kinase CK2 took place in Homburg, Germany, from 6 September to 9 September 2016. Over 80 scientists from Australia, China, Japan, USA, Canada, Denmark, France, Italy, Spain, Poland and Germany participated. After the opening lecture by Lorenzo A. Pinna, Padova, Italy, entitled Exploring the CK2 Paradox: Restless, Dangerous, Dispensable, the scientists reported their latest research on the structural characterization of CK2, hence leading directly to the development of CK2 inhibitors. The driving force behind the development of inhibitors is their use in the treatment of various diseases, which was the next topic of the conference. New findings on protein kinase CK2 were addressed in the following session. The final topic of the conference addressed the role of CK2 in differentiation and development.

Lorenzo A. Pinna reminded everybody that the original name “casein kinase” was changed to protein kinase CK2 nearly 20 years ago, because casein did not appear to be among the natural substrates of CK2 [1]. Furthermore, CK2 has now been validated as a “druggable” kinase, which is targeted, in particular, in cancer cells. The most exciting news in this field was the establishment of viable CK2α^−/−^,α’^−/−^ cells by CRISPR/cas9 technology and the subsequent phosphoproteome analysis of these non-neoplastic CK2α^−/−^,α’^−/−^ cells.

Various crystallographic structures of the full-length as well as truncated version of CK2α are already published. The group of Karsten Niefind analysed CK2α and CK2α’ in complex with ATP-competitive inhibitors [2]. This structural information not only provided further information about the ATP binding pocket, the interaction of individual residues in the polypeptide chain of both catalytic subunits with the inhibitors, and structural deformation of the enzymes, but also offered new elements for the design of more selective and potent inhibitors.

Starting approximately at the beginning of this century, there is an increasing list of inhibitors of CK2 comprising different chemical entities. Some of these are natural products, or compounds identified in drug libraries as well as molecules identified by an in silico approach, followed by chemical synthesis. Georgio Cozza compiled these different approaches to find more potent inhibitors of human CK2 [3]. Some of these approaches are based on crystallographic information about CK2 alone or in complex with an inhibiting compound. History has shown that inhibitors, which were considered initially to be CK2 specific, later on turned out to target multiple kinases. It is now clear that they need to be tested on a large scale of different kinases, before selectivity and specificity can be evaluated. Moreover, there is an increasing need for the analysis of off-target effects. Most of the inhibitors of CK2 known so far are indeed ATP-competitive inhibitors. 

Samer Haidar reported on the development of new inhibitors based on an indeno[1,2-*b*]indole scaffold which was already known as a lead structure for the development of human CK2 inhibitors [4]. By using a pharmacophore model, which was developed on the inhibition data obtained with CK2 and more than 50 indeno[1,2-*b*]indoles, for mining the ZINC compound database, the natural compound bikaverin was identified and turned out to be a new potent CK2 inhibitor. 

The group of Emilio Itarte used the inhibitor CX-4945, which is orally available, either alone or in combination with other cytostatic drugs for the treatment of cancer [5]. They used CX-4945 in combination with temozolomide to treat mice affected by glioblastoma. The best survival rates were obtained with a metronomic therapy comprising a combination of temozolomide and CX-4945 every 6 days. The group of Barbara Guerra used 1,3-dichloro-6-[(*E*)-((4-methoxyphenyl) imino)methyl]dibenzo(b,d)furan-2,7-diol (D11) also as a CK2 inhibitor for the treatment of glioblastoma cells [6]. They found that D11 led to a destabilization of HIF-1α under hypoxic conditions. They conclude from their results that D11 treatment deprives glioblastoma cells of oxygen and nutrient supply. These properties may improve the therapy of glioblastoma in combination with other cytostatic components.

The group of Joachim Jose established a bacterial surface display 12mer peptide library and screened it with fluorescence labelled human CK2 [7]. By this strategy, new non-ATP competitive CK2 inhibitors were identified. The most potent inhibitor identified, B2, not only inhibited CK2 kinase activity by binding to an allosteric pocket, but also disturbed the interaction of CK2α with CK2β.

Some years ago, the group of Claude Cochet and Odile Filhol published findings on a cyclic peptide interfering with the CK2 subunits interaction [8]. As a further development, a cell-permeable version of this peptide was analysed. This construct turned out to inhibit the cellular association of CK2α and CK2β, and consequently led to a shift in the phosphorylation efficiencies, in particular of CK2β-depending substrates. Moreover, they could show that the peptide led to a dissociation of already formed CK2 holoenzymes.

It is still an enigma how CK2 can regulate multiple functions and pathways in a cell without being itself regulated. By systematic analyses of published literature and proteomics databases together with the computational assembly of networks of CK2, the Litchfield group found that by phosphorylating its substrates [9], CK2 can modulate other post-translational modifications and, vice versa, that post-translational modifications affect CK2 phosphorylation of substrates.

Besides inhibitors, another approach was exploited for the study of CK2 functions, namely knock-down of expression of CK2 subunits. By this approach, the group of Hashemolhosseini found reduced activity of nicotinamide adenine dinucleotide dehydrogenase and succinate dehydrogenase in CK2β-deficient muscle fibres [10]. This result indicates a role of CK2β in the regulation of the oxidative capacity of skeletal muscle fibres.

Full or conditional CK2 knock-out mice were used for the elucidation of the role of CK2 in brain development, neuronal activity and behaviour, which was summarised by the group of Heike Rebholz [11]. They further described brain-specific substrates and the role of CK2 in various brain diseases such as glioblastoma, Parkinson’s disease, Huntington’s disease, Amyotrophic Lateral Sclerosis (ALS) and Alzheimer’s disease.

The role of CK2 in the development of Drosophila was addressed by the group of Ashok Bidwai [12]. This article focuses on the structure, subunit diversity and mutations in the CK2 subunits and in particular on the role of CK2 in eye development.

The role of CK2 in the adipogenic differentiation of mesenchymal stem cells was addressed by Lisa Schwind and her colleagues [13]. CK2 seems to be essential at early time points after the start of differentiation, where it influences cell proliferation and the expression of different transcriptional factors such as C/EBPα and PPARγ2.

Adam Johnson and Ming Wu reported on the role of CK2 in the regulation of metal ion transport in *Saccharomyces cerevesia* and in mammalian cells [14]. On one hand, divalent metal ions such as Mg^2+^, Mn^2+^ and Co^2+^ are required for CK2 activity. On the other hand, CK2 seems to be responsible for the toxicity of Al^3+^ and As^3+^ ions. Furthermore, CK2 subunits are implicated in metal ion transport. In the case of Zn^2+^ ions, it was shown that CK2 phosphorylates the Zinc ion channel ZIP7, the epithelial Na^+^ channel and the chloride channel CFTR.

Another topic of the CK2 meeting was the search for new substrates and new functions of CK2. One of the substrates of CK2, particularly in pancreatic β-cells, is the transcription factor PDX-1. The group of Claudia Götz showed that the CK2 phosphorylation sites on the polypeptide chain of PDX-1 interfere with the binding site for PCIF1, which is an E3 ubiquitin ligase adaptor protein [15]. Binding of PCIF1 to CK2-phosphorylated PDX-1 promotes the degradation of PDX-1.

The group of Kubinski and Maslyk reported on the interaction of CK2 with an atypical kinase, Rio1, which plays a role in cellular proliferation and ribosome biosynthesis [16]. Rio1 is not only a substrate for CK2 but also a binding partner. At least in yeast, Rio1 is one of the very few binding partners of CK2α, as shown by an immunoprecipitation. A number of benzimidazole-derived compounds inhibited both, CK2 and Rio1. Molecular docking approaches indicated that the inhibitor tetrabromobenzimidazole (TBB) binds to the ATP-pocket of both kinases in quite a similar manner.

A topic addressed in two reviews was the role of CK2 as a target for the treatment of cancer cells. The group of Khalil Ahmed summarized their strategy for the delivery of RNAis down-regulating CK2 in cancer cells using nano-capsules [17]. In order to target cancer cells specifically, tenfibgen was used as a ligand of tenascin-C receptors, which are particularly numerous on the surface of cancer cells. This strategy proved successful with cancer cell lines and in animal models, opening the window for its use in the therapy of various tumours.

The group of Isabel Dominguez used a more general approach to analyze the implications of CK2 in cancer, its influence on cellular signalling pathways and its potential as a target in anti-cancer cells therapy [18]. The general conclusion is that CK2 is overexpressed in many cancers and this overexpression is accompanied by elevated protein kinase activity. Knock-down of individual subunits of CK2 resulted in the perturbation of diverse signalling pathways, including the SMAD2/3, β-catenin, PI3K-Akt-mTOR, and IκB-NFκB pathways and signalling pathways regulating apoptosis. The influence of CK2 on these pathways is qualitatively and quantitatively different in diverse tumours.

This 2016 CK2 conference emphasized once more that protein kinase Ck2 is an enticing enzyme with a broad range of physiological functions. The most unexpected new finding was that life without CK2—at least for non-neoplastic animal cells in culture—seems to be possible under special conditions. Furthermore, the role of CK2 in controlling cell proliferation, development and differentiation has been further elucidated. Because it appears to influence a variety of different cellular signalling pathways, the effects of CK2 inhibitors need to be considered under this light and careful use of such compounds is mandatory. In summary, the results presented during the 8th International Conference on Protein Kinase CK2 in Homburg underlined CK2’s status as an emerging drug target and strengthened the view that pharmacological down-regulation of human CK2 with small molecules is a promising approach, not only in cancer.
